# The role of protein kinase C in diabetic microvascular complications

**DOI:** 10.3389/fendo.2022.973058

**Published:** 2022-08-17

**Authors:** Deng Pan, Lin Xu, Ming Guo

**Affiliations:** ^1^ Xiyuan hospital of China Academy of Chinese Medical Sciences, Beijing, China; ^2^ National Clinical Research Centre for Chinese Medicine Cardiology, Xiyuan Hospital of China Academy of Chinese Medical Sciences, Beijing, China; ^3^ Graduate School of Beijing University of Chinese Medicine, Beijing, China; ^4^ Gynecological Department of Traditional Chinese Medicine, China-Japan Friendship Hospital, Beijing, China

**Keywords:** protein kinase C, diabetes mellitus, diabetic microvascular complications, microvascular, review

## Abstract

Protein kinase C (PKC) is a family of serine/threonine protein kinases, the activation of which plays an important role in the development of diabetic microvascular complications. The activation of PKC under high-glucose conditions stimulates redox reactions and leads to an accumulation of redox stress. As a result, various types of cells in the microvasculature are influenced, leading to changes in blood flow, microvascular permeability, extracellular matrix accumulation, basement thickening and angiogenesis. Structural and functional disorders further exacerbate diabetic microvascular complications. Here, we review the roles of PKC in the development of diabetic microvascular complications, presenting evidence from experiments and clinical trials.

## Introduction

Diabetes mellitus (DM) is widespread globally, and the complications of diabetes are the main causes of morbidity and mortality (1, 2). In patients with DM, hyperglycemia is a major risk factor for the development of microvascular complications (3). Long-term DM affects the function and structure of blood vessels, causing diabetic macrovascular and microvascular complications. Diabetic nephropathy, diabetic retinopathy and diabetic peripheral neuropathy are thought to be the main types of diabetic microvascular complications (4). According to the Outcome Reduction with an Initial Glargine Intervention (ORIGIN) trial, for patients with a baseline HbA_1c_ of more than 6.4%, interventions to normalize normal fasting glucose significantly reduced the risk of diabetic microvascular complications (5). In patients with type 2 diabetes mellitus (T2DM), antihyperglycemic therapy also demonstrated a favorable effect in reducing the risk of microvascular complications (6). However, despite glucose-lowering therapy, a high residual risk of diabetic microvascular complications remains in patients with DM (7). In addition, the interaction of genetic, environmental and metabolic factors is involved in microvascular complications. Thus, there is still an unmet need for a better understanding of the pathogenesis of diabetic microvascular complications (8).

Protein kinase C (PKC) participates in various cellular responses associated with DM (9). Activation of PKC is significantly increased under conditions of elevated glucose levels (22 mM) *in vitro* (10). Hyperglycemia triggers the glycolysis pathway and further enhances the synthesis of diacylglycerol (DAG), which activates PKC (11). Activation of PKC then facilitates activation of NADPH oxidase, after which the redox reaction starts and redox stress accumulates. As a consequence, reactive oxygen species (ROS) and advanced glycation end products (AGEs) accumulate in cells, leading to cell death and apoptosis (12, 13). The promotion of redox stress is observed in various vascular cells, including pericytes, endothelial cells, smooth muscle cells, podocytes and others (14). Therefore, vascular dysfunction generated by the aforementioned mechanism promotes diabetic microvascular complications, which mainly influence alterations in blood flow (15), extracellular matrix synthesis and basement membrane thickening (16, 17), vascular permeability (18), and angiogenesis (19). In this review, we focus on the roles of PKC and PKC isoforms in the development of diabetic microvascular complications.

## PKC: classification and structures

**Figure 1:**
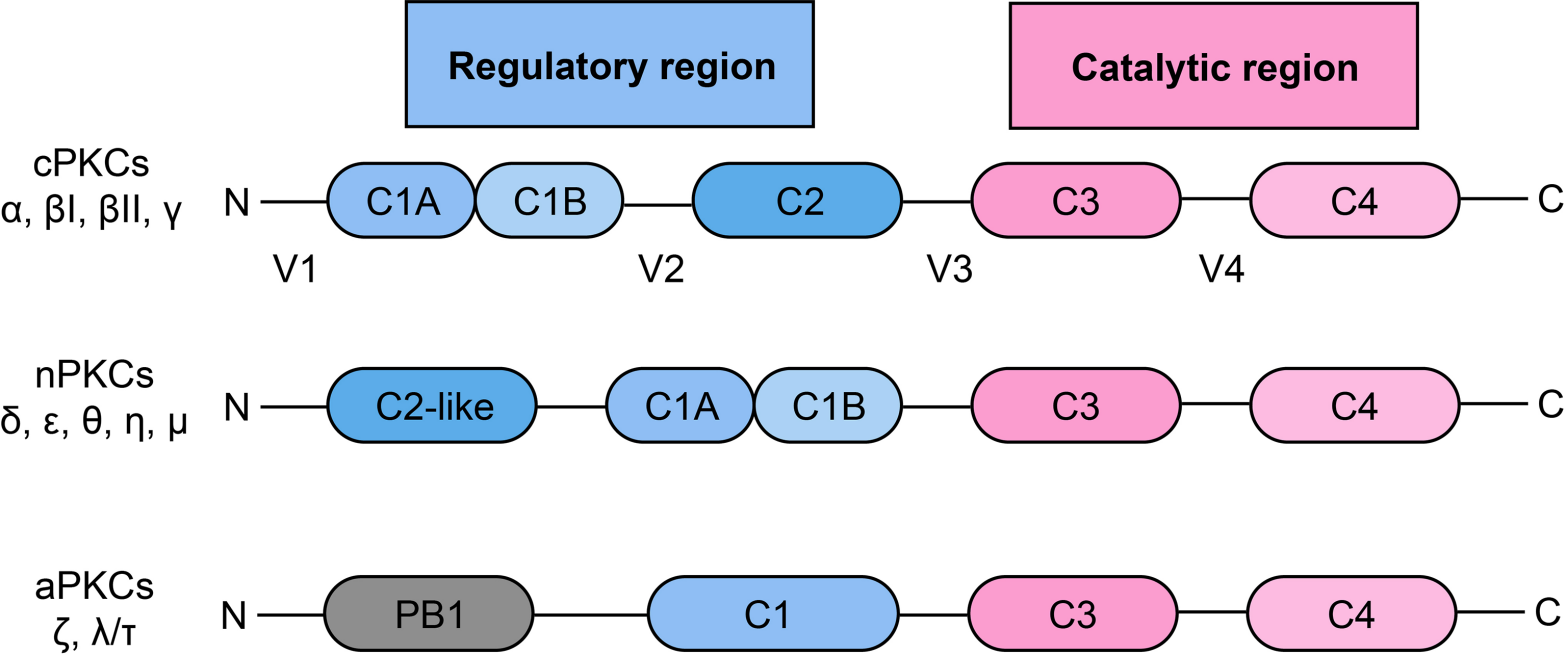
Classification and structural of protein kinase C.PKC: protein kinase C; cPKC: conventional protein kinase C; nPKC: novel protein kinase C; aPKC: atypical protein kinase C.

PKC is a family of serine/threonine protein kinases, which was discovered over 30 years ago (20). According to the pattern of activation, PKC can be divided into three subgroups, including classical/conventional PKCs (cPKCs): PKC-α, βI, βII, γ; novel PKCs (nPKCs): PKC-δ, ϵ, θ, η, μ; atypical PKCs (aPKCs): PKC-ζ, λ/τ (21, 22) (**Figure 1**). The activation of cPKCs requires both calcium and DAG or phorbol esters, while nPKCs are activated by DAG or phorbol esters but not by calcium. The aPKCs are not activated by either calcium or DAG/phorbol esters. aPKCs are regulated by protein–protein interactions through the PB1 domain (22, 23). All of the PKC isozymes comprise an N-terminal regulatory region and a C-terminal catalytic region, and the two regions are linked by a V3 hinge (24). In cPKCs and nPKCs, the regulatory region contains the C1 domain, which can be further divided into the tandem C1A and C1B subdomains, which are duplicates and are rich in cysteine sequences (25). In addition, cPKCs contain a C2 domain, which is able to bind to calcium. However, this C2 domain is not present in nPKCs, which have a C2-like domain (cannot bind to calcium) instead. Therefore, nPKCs are not activated by calcium (26). Once the C1 domain binds to DAG, a pseudosubstrate is removed from the catalytic domain, leading to enzyme activation (25, 26).

## Pathogenesis and the role of PKC in diabetic microvascular complications

### Diabetic nephropathy

Diabetic nephropathy (DN) is characterized by proteinuria and a decline in glomerular filtration rate (GFR). If DN is untreated, the resulting end-stage renal failure and uremia are fatal (27). Various kinds of renal cells, including podocytes, endothelial cells, smooth muscle cells, mesangial cells and inflammatory cells, are affected under hyperglycemia (28). Hemodynamic changes play an important role in the pathogenesis of DN, which arises from vasoactive factor release, signal transduction and metabolic changes under hyperglycemic conditions. In the early stage of DN, increased plasma flow and glomerular capillary pressure cause an increase in GFR, termed hyperfiltration (29). Hyperfiltration also precipitates thickening of the glomerular basement membrane (GBM) (hyperplasia) and the detachment, loss, and death of podocytes (30). Moreover, renin and angiotensin-II synthesis in mesangial cells were found to be promoted under high-glucose conditions, which further gave rise to an increase in glomerular capillary pressure, microvascular permeability, and renal cell proliferation (31, 32). As a result, proteinuria and glomerulosclerosis were substantially induced (33). Moreover, with the feedback of hypertrophy and hyperplasia, the proximal tubule length significantly increases, leading to an increased reabsorption of glucose, fatty acids, amino acids, growth factors and cytokines (34). This metabolic production in turn leads to energetic imbalance, such as redox abnormalities and fibrosis. The aforementioned pathological processes ultimately result in extracellular matrix (ECM) deposition in the tubules, which is deemed to be the major determinant in the development of DN (35). Consequently, the GFR is eventually decreased owing to progressive glomerular and tubular injury (36).

**Figure 2:**
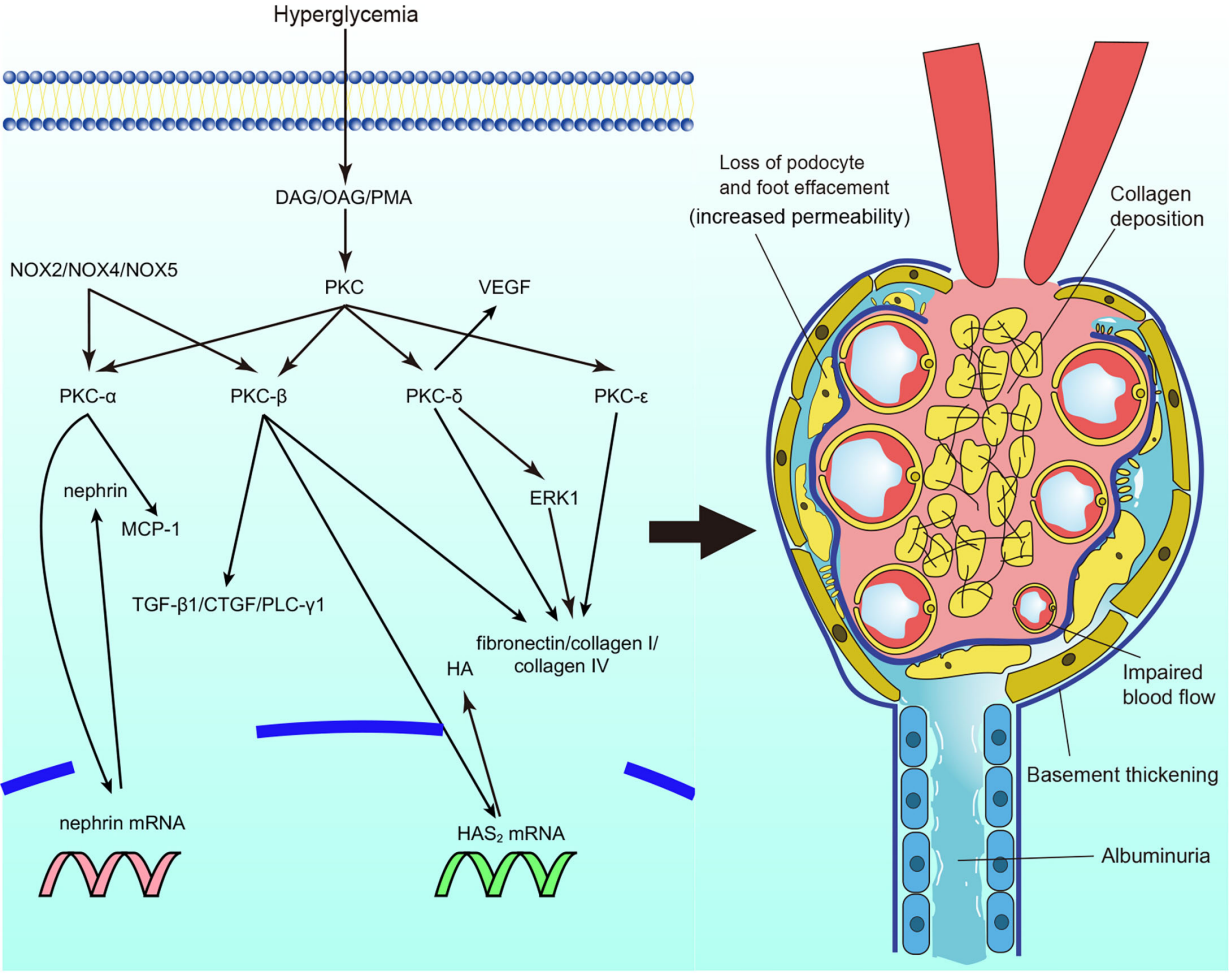
Protein kinase C (PKC) in diabetic nephropathy. PKC activation in diabetic nephropathy impairs glomerular blood flow and filtration, leads to albuminuria, causes extracellular matrix accumulation and collagen deposition particularly. DAG, diacylg lycerol; OAG, oleoyl acetyl glycerol; PKC, protein kinase C; MCP-1, monocyte chemoattractant protein-1; TGF-β1, transforming growth factor β1; CTGF, connective tissue growth factor; PLC-γ1, phospholipase Cγ1; HAS2, Hyaluronan synthase 2; HA, hyaluronan; ERK, extracellular signal-regulated kinase; VEGF, vascu lar endothelial growth factor; NOX, nicotinamide adenine dinucleotide phosphate oxidase.

The activation of PKC is involved in microvascular contraction within the kidney, affecting the function of glomeruli and contributing to the progression of diabetic nephropathy (**Figure 2**). The microvascular contraction of glomeruli is enhanced under high-glucose conditions, and blood flow is consequently decreased. This is mainly caused by changes in the levels and sensitivity of contraction factors. Under high-glucose conditions, the levels of vascular contraction factors, such as prostaglandin E2 and prostaglandin F2 alpha, are elevated (15). In addition, the sensitivity of vascular contraction factors is increased in diabetic nephropathy. In diabetic mice, phenylephrine-induced interlobar artery (ILA) contraction was significantly enhanced, which caused ILA dysfunction. ILA dysfunction further reduced blood flow in the glomerulus and induced the progression of diabetic nephropathy. Treatment with rottlerin, a calcium-independent protein kinase C (PKC-δ) inhibitor, alleviated basal overcontraction (37, 38). Similar results were observed in diabetic Zucker rats; the nonselective PKC agonist phorbol-12,13 dibutyrate (PDBU) significantly reduced renal cortical blood flow and increased the mean arterial pressure (39). The results indicate that PKC activation is associated with decreased blood flow and increased renal perfusion pressure, causing glomerulosclerosis and reducing glomerular filtration.

Impaired barrier function is associated with the deterioration of diabetic nephropathy, during which proteinuria is usually observed. The role of PKC in the vascular permeability of diabetic nephropathy mainly focuses on the glomeruli. Nephrin is one of the most well-studied proteins involved in vascular permeability. Nephrin is essential for podocytes to sustain the integrity of the slit diaphragm to maintain filtration (40, 41). The expression levels of nephrin mRNA and protein were significantly reduced in streptozotocin (STZ)-induced DM mice. However, in global PKC-α knockout mice, nephrin expression was unaffected in the mice with DM (42). Thallas-Bonke et al. also found that PKC-α and PKC-β were overexpressed in DM mice, whereas deletion of NOX4 (nicotinamide adenine dinucleotide phosphate oxidase) reversed the overexpression of PKC-α and PKC-β, with normalized nephrin expression observed (43). In addition, mediation of RhoA downstream of C3aR in endothelial cells by PKC resulted in kidney damage and increased blood vessel permeability (44). Moreover, VEGF plays an important role in increased capillary permeability. Diabetic mice treated with calphostin C showed reduced expression of VEGF in their mesangial cells compared to mice that were not treated with calphostin C (45). Mima et al. explored the interaction of PKC-δ and VEGF in diabetic nephropathy. The expression of VEGF was induced under high-glucose conditions, whereas loss of VEGF signaling was observed, which indicated VEGF resistance. Notably, VEGF signaling glomerular endothelial was not inhibited in global PRKCD knockout mice. In addition, less extracellular matrix and albuminuria were also observed (46). The results suggest that activation of different PKC isoforms is involved in increased vascular permeability and that PKC-α and PKC-β are interesting molecules in the maintenance of glomerular filtration function. PKC-δ may play an important role in the regulation of VEGF expression and prevention of resistance to VEGF in the glomeruli.

Structural abnormalities are the most well-studied pathology in diabetic nephropathy. It has been shown that expansion of the basement membrane and ECM accumulation are induced by high-glucose conditions (47). The synthesis of ECM proteins, such as fibronectin, laminin, and types I, III, and IV collagen, was found to be increased in renal glomerular mesangial cells, accompanied by upregulated activation of PKC (48–50). PMA (PKC activator) and the cell-permeable DAG analog oleoyl acetyl glycerol (OAG) also mimicked these effects (48). Treatment with the PKC inhibitor chelerythrine reduced the expression and synthesis of fibronectin and prevented basement membrane thickening (51). In addition, elements of the ECM regulated by activation of PKC have also been studied. Hyaluronan (HA), a key element of the ECM, was incrementally secreted under hyperglycemia, and inhibition of PKC-β reduced the expression of HAS_2_ (hyaluronan synthase 2) mRNA and secretion of HA (52). A general PKC inhibitor also reversed the hyperglycemia-induced elevated synthesis of collagenous and total ECM proteins and decreased gelatinase activity in endothelial cells (53). The results suggest that PKC activation is involved in the synthesis of ECM proteins and degradation of the ECM in diabetic nephropathy. Furthermore, studies on the activation of PKC isoforms in DN have also been conducted. PKC-α and PKC-β activation was increased in mesangial cells under high concentrations of glucose, and the synthesis of fibronectin and IV collagen paralleled their activity (54, 55). LY333531 (a specific PKC-β inhibitor) prevented the hyperglycemia-induced expression of ECM components in mesangial cells (55). The same results were observed in global PKC-α/PKC-β double knockout mice, accompanied by alleviation of the development of albuminuria (56). However, despite a reduction in the albumin/creatinine ratio, the ratio was not completely reversed (56). Tokuyama et al. found that a PKC-β inhibitor might enhance reductions in mesangial cell (3) H-thymidine and (3) H-proline incorporation, which reduced the synthesis of collagen by mesangial cell (57). The nPKCs PKC-δ and PKC-ϵ also participate in ECM protein synthesis. The synthesis of ECM proteins in mesangial cells was reduced in PKC-δ^-/-^ and PKC-ϵ^-/-^ STZ-induced DM mice and in mice with exogenous PKC-δ inhibition (46, 58, 59). Interestingly, PKC-ϵ^-/-^ mice did not show a profibrotic phenotype in any organ other than the kidney (58). Of note, Baccora et al. reported that activation of PKC-δ, rather than PKC-α or PKC-β, was increased in mesangial cells under high-glucose conditions (59). The membrane association of PKC-ζ was confirmed to be prevented by rosiglitazone, along with decreased expression of collagen IV in cultured mesangial cells (60). Nevertheless, the role of aPKCs still needs more exploration. Moreover, other fibrotic factors and pathways that interact with PKC are discussed. Transforming growth factor β1 (TGF-β1) is one of the most important factors that causes basement thickening and ECM accumulation in diabetic nephropathy (61). PKC activation and the expression of TGF-β1 in mesangial cells and glomerular endothelial cells were increased under treatment with glycated albumin and advanced oxidation protein products (the level of glycated albumin and advanced oxidation protein products are elevated under high glucose, which mimics the alternations *in vivo*) (62–64). Inhibition of PKC-α and PKC-β attenuated the expression of TGF-β1 and connective tissue growth factor (CTGF) in glomerular endothelial cells and mesangial cells (54, 55, 62, 64–66). Interactions between other molecules and PKC in terms of ECM protein synthesis and basement thickening have also been explored. Global Akr1b3 knockout mice showed inhibition of PKC activation, with reduced ECM accumulation and glomerular hypertrophy in renal cortical tissue (67). The homologous genes NOX2, NOX4, and NOX5 were also found to inhibit PKC-α and PKC-β activation, followed by decreases in hyperglycemia-induced ROS and expression of TGF-β1 and monocyte chemoattractant protein-1 (MCP-1), which prevented ECM accumulation and basement membrane thickening in mesangial cells and podocyte, and thus decreased albumin excretion (68–70). Extracellular signal-regulated kinase (ERK) is also involved in ECM accumulation. PKC-δ expression was increased in the membranous fraction under high-glucose conditions. Treatment with the PKC-δ inhibitor rottlerin, but not the cPKC inhibitor Gö6976, abrogated ERK expression and decreased hyperglycemia-induced responsiveness to TGF-β1 in mesangial cells, blocking the fibrotic response (71). In contrast, Tuttle et al. discovered that PKC-β inhibition caused decreased expression of ERK1 and ERK2 in mesangial cells (72). Moreover, Wu et al. reported the involvement of phospholipase C γ1 (PLC-γ1) in the development of diabetic nephropathy. PLC-γ1 inhibition suppressed PKC-β-induced protein kinase B (Akt) S473 phosphorylation in mesangial cells under high-glucose conditions, and collagen I upregulation was also prevented by the PLC-γ1 inhibitor U73122 (73). In summary, current studies indicate that overexpression of PKC is involved in the accumulation of the ECM, and mesangial cells account for most of the ECM accumulation; however, regarding PKC isoforms, some studies have shown contradictory results (56, 57, 59). The results may be influenced by the duration of cell culture. The authors suggest that in the development of diabetic nephropathy, specific PKC isoforms may participate in the early process of exacerbation. However, with the development of diabetic nephropathy, more PKC isoforms may be involved in its pathogenesis when high-grade proteinuria occurs. Consequently, it is also vital to explore different PKC isoforms in various types of cells in the kidney and stages of diabetic pathogenesis. In addition, VEGF plays an important role in the development of diabetic nephropathy, the role of VEGF in ECM accumulation may also be a promising topic to be explored in the future.

### Diabetic retinopathy

**Figure 3:**
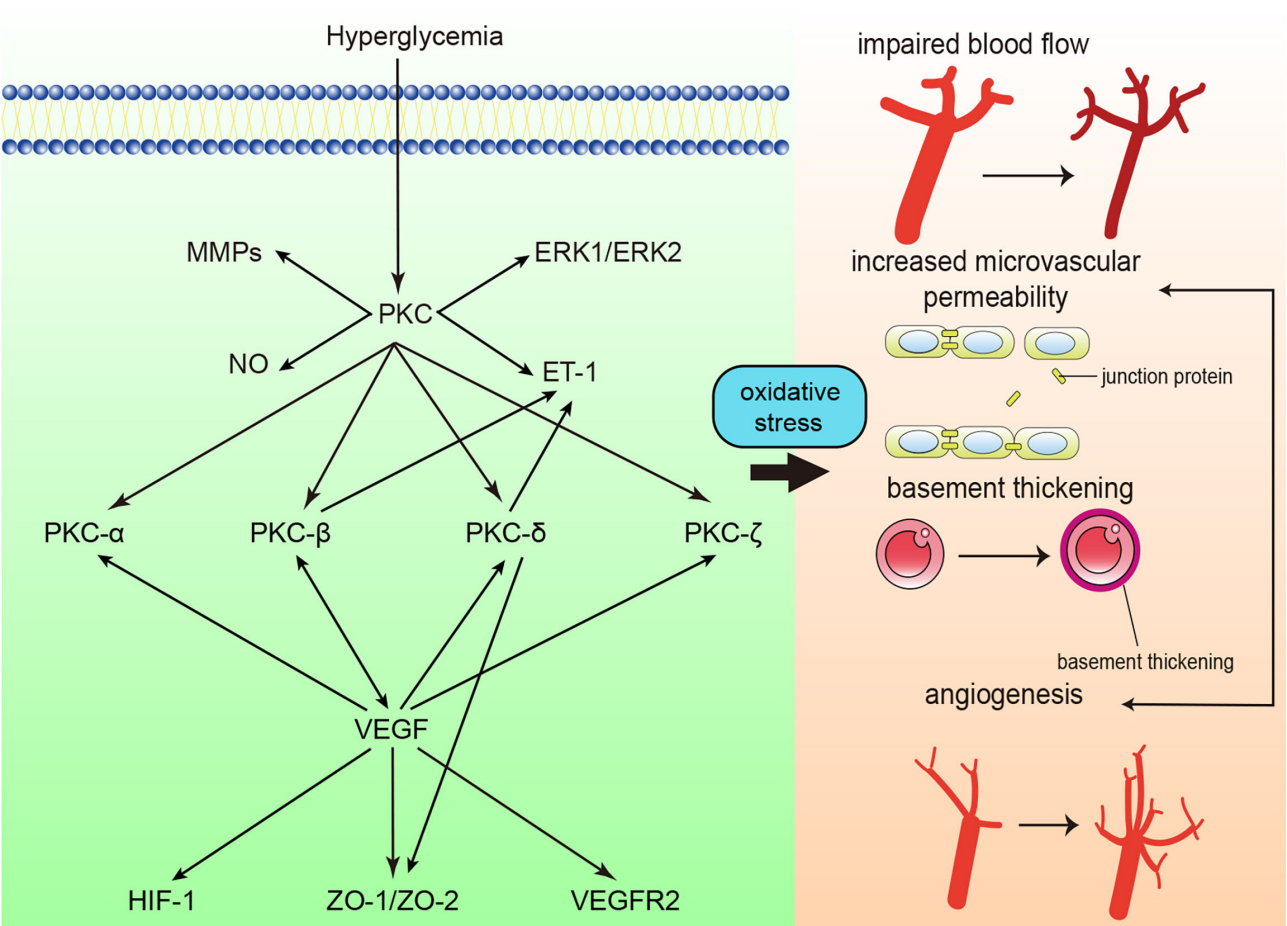
Protein kinase C (PKC) in diabetic retinopathy. PKC activation in diabetic retinopathy influences blood flow, causes retinal microvascular constriction, induces angiogenesis and increases microvascular permeability. ET-1, endothelin-1; NO, nitric oxide; MMP, matrix metalloproteinase; ERK, extracellular signal-regulated kinase ; VEGF, vascular endothelial growth factor; VEGFR2, vascular endothelial growth factor receptor 2; ZO, zonula occludens; HIF-1, Hypoxia-inducible factor 1.

Oxidative stress is considered an important factor in the pathogenesis of diabetic retinopathy (DR), and accumulated reactive oxygen species ultimately result in the impairment of retinal tissue and vessels. PKC activation is one of the main factors involved in accumulated oxidative stress (74, 75). High glucose concentrations cause damage to the function and structure of the retina, including loss of pericytes, increased vascular permeability, thickening of the retinal capillary basement membrane, tissue ischemia, etc. (**Figure 3**) (74). The processes mentioned above give rise to the initial stage of DR, termed nonproliferative diabetic retinopathy (NPDR). In the NPDR stage, visual impairment is not always noticed, whereas sight-threating DR and blindness are preventable (76). However, if NPDR is left untreated, vascular endothelial growth factor (VEGF) protein levels are upregulated due to ischemia/hypoxia through hypoxia-inducible factor 1 (HIF-1) activation (77). Upregulation of VEGF causes the formation of new blood vessels, which is called neovascularization. This drives DR into the proliferative diabetic retinopathy (PDR) stage. In this phase, newly formatted permeable vascular tufts and macular edema appear and ultimately cause loss of vision (78, 79).

Activation of PKC mediates the progression of decreased retinal blood flow in DM. The mean circulation time (MCT) in diabetic rats was significantly increased. In contrast, when the rats were treated with intravitreal injection of LY333531, the MCT was shortened to half the MCT of those that were not treated (80). PKC-mediated inflammation may be one of the factors of decreased blood flow. In the retinal microcirculation of diabetic rats, an increased number of leukocytes were trapped, leading to blood flow disturbances in the retinal microvasculature. LY333531 significantly reduced the number of trapped leukocytes and increased the retinal blood flow of diabetic rats (81). In addition, the activation of PKC may cause the overexpression of endothelin-1 (ET-1), which can also explain the decrease in retinal blood flow. The expression of ET-1 and activation of PKC were increased in parallel by 2-fold in bovine retinal endothelial cells (BRECs) under high-glucose conditions. The overexpression of ET-1 was inhibited by the general PKC inhibitor GF109203X. It was further discovered that PKC-β and PKC-δ were significantly overexpressed in membranous fractions, which indicated that the activation of PKC-β and PKC-δ mediated ET-1 expression (82). Moreover, nitric oxide (NO) insufficiency participates in the alteration of retinal blood flow. The synthesis of NO from retinal microvascular endothelial cells (RMECs) was downregulated under high-glucose conditions, whereas a PKC inhibitor increased the accumulation of NO and restored the blood flow in the retina (83). PKC-β and PKC-δ are two main isoforms that have been studied regarding blood flow in the retina, and studies on other isoforms are in progress.

It has also been proven that retinal vascular permeability is increased under high-glucose conditions, along with levels of reactive oxygen species (84, 85). The application of a PKC inhibitor reduced high glucose-induced albumin leakage (85). VEGF is mainly studied in terms of retinal vascular permeability. The expression of PKC-β and VEGF were both significantly increased under high-glucose conditions, and the inhibition of PKC-β and PKC-β mutant mice showed attenuated vascular barrier function and less albumin leakage (86–88). PKC-β activation induced occludin Ser490 phosphorylation, which led to the ubiquitination required for VEGF-induced permeability and exacerbated retinal barrier dysfunction (89). In addition to PKC-β, activation of PKC-α, PKC-ζ, PKC-δ and aPKC induced by VEGF is involved in impaired tight junctions and activation of inflammatory pathways and causes hyperpermeability (90–94). PKC-β, PKC-α, and PKC-δ levels were found to be increased in membranous fractions. However, treatment with a PKC-β inhibitor rescued 95% of the VEGF-induced increase in vascular permeability, indicating that PKC-β plays a major role in VEGF-induced hyperpermeability in diabetic retinopathy (90, 91). Moreover, studies focusing on gap junctions were also conducted. Gap function is impaired due to the loss of zonula occludens (ZO)-1, ZO-2, etc. Inhibition of PKC-δ blocked translocation from the cytosol to the membrane and restored the loss of gap junction proteins (95). However, PKC was not found to participate in claudin-1 (another gap junction protein) expression (96). In summary, PKC-β is thought to be the most crucial PKC isoform that participates in increased retinal microvascular permeability, and most related clinical trials have also focused on PKC-β (see below). The role of PKC in specific gap junction proteins still needs further exploration. PKC is also promising for detecting structural impairment in cell junctions visually.

Basement thickening is also one of the most common features in the early stage of diabetic retinopathy (95). As mentioned above, PKC-β overexpression was observed in STZ-induced rats. Treatment with hesperetin (Hsp) suppressed PKC-β expression, abolished DM-induced retinal capillary basement membrane thickness, and prevented retinal microvascular dysfunction (88). Giordo et al. reported an association between PKC activation and endothelial-to-mesenchymal transition (EndMT). In high glucose-exposed human retinal endothelial cells, the general PKC inhibitor chelerythrine significantly reduced ROS levels and EndMT. Moreover, the researchers also observed attenuated fibrotic processes (97). However, due to the correlation between structural and functional changes in diabetic retinopathy (98), more studies on the association between PKC activation and ECM augmentation are expected.

Activation of PKC enhanced angiogenesis in diabetic retinopathy. As mentioned above, PKC activation enhances the expression of VEGF in the retina. Nonetheless, vascular permeability is increased, which exacerbates the development of diabetic retinopathy. The activation of PKC accelerates migration, tube formation, and cellular proliferation in diabetic retinopathy, and the PKC-mediated upregulation of extracellular signal-regulated kinase (ERK) 1/2 and matrix metalloproteinase (MMPs) may account for the enhanced angiogenesis (99, 100). Although enhanced angiogenesis is observed in the retina under high-glucose conditions, the newly formed microvasculature shows impaired function, which accelerates the progression of diabetic retinopathy.

### Diabetic neuropathy

Diabetic neuropathy is a syndrome that affects the peripheral nervous system, and both the somatic and autonomic divisions are impaired. It is mainly thought to be the result of thickening of the capillary basement membrane and endothelial hyperplasia, as mentioned above (101, 102). Sensory axons, autonomic axons and, rarely, motor axons are mainly influenced in diabetic neuropathy. Moreover, the spinal cord is damaged in diabetic neuropathy (31, 103, 104). Damage to sensory axons usually causes symptoms that are easy to notice, such as hyperalgesia, paresthesia, and allodynia. Notably, the loss of nerve conduction velocity and nerve terminals tends to occur in long nerve fibers, which is called a fiber length-dependent pattern. Thus, numbness, nighttime pain, dysesthesia, and sensory loss always appear on the feet and then ascend to the hands, which is called the ‘stocking and glove pattern’ distribution (105). In addition, autonomic axons are also affected. In patients with DM, several autonomic nervous system symptoms can be observed. One of the most common symptoms is orthostatic hypotension, and the autonomic nervous system fails to regulate heart rate and blood flow; consequently, upright position blood pressure cannot be well maintained (106). Other autonomic nervous system symptoms, including gastroparesis, nausea, bloating, and diarrhea, should be considered evidence of the development of diabetic neuropathy. In addition, symptoms such as depression and sleep disturbance should be differentiated and emphasized (107). Motor neurons are located inside the blood–brain barrier, which may explain why damage to motor axons is rarely observed (108). The symptoms of diabetic neuropathy mostly refer to motor dysfunction, including falling, body sway, and dorsiflexion of fingers or toes (109, 110).

Studies concerning activation of PKC in diabetic neuropathy mainly focus on blood flow and conduction velocity. The blood flow in sciatic motor and saphenous nerve sensory were significantly decreased in diabetic rats, along with higher response to noxious mechanical and thermal stimuli. What’s more, NO synthase was also found to be inhibited in diabetic rats. LY333531 treatment attenuated the impaired blood flow and NO synthase activity (111). WAY151003 (general PKC inhibitor) and cremophor (an agent that prevents PKC activation) were also found to be effective in improving motor conduction velocity and correcting blood flow deficits in the sciatic motor and saphenous areas (112, 113). In addition, basement membrane thickening is associated with nerve damage in patients with diabetic neuropathy (114). Nonetheless, as a small number of studies have been conducted on microvascular functional and structural damage in diabetic neuropathy, addressing microvascular problems is thought to be an adjunct therapy in treating diabetic neuropathy.

## Clinical trials of PKC inhibition in diabetic microvascular complications

As mentioned above, *in vivo* and *in vitro* experiments have demonstrated that various PKC isoforms participate in the development of diabetic microvascular complications. Since different general and specific PKC inhibitors have been explored, clinical trials have also been conducted to evaluate the efficacy and safety of PKC inhibitors in patients with diabetic microvascular complications (**Table 1**). One clinical trial showed that the general PKC inhibitor PKC412 reduced macular edema and improved visual acuity. However, gastrointestinal side effects (diarrhea, nausea, and vomiting) were associated with PKC412, along with dose-related liver toxicity, indicating that general PKC inhibitors may not be an appropriate choice for clinical application due to their side effects (115).

**Table 1:** Clinical trials of PKC inhibitors on diabetic microvascular complications.

Author Year	Participants(n)	Intervention group (n)	Placebo group (n)	Inclusion criteria	Intervention	Dose&Duration	Results	Reference
Campochiaro 2004	141	32 (50mg/d) 38 (100mg/d) 37 (150mg/d)	34	18-85 years of age; diagnosis of type 1 or type 2 diabetes mellitus with nonproliferative diabetic retinopathy or no more than mild proliferative diabetic retinopathy, as defined by ETDRS level 61; retinal thickening in the study eye within 3000 μm of the foveal center with an area of at least 0.5 disc areas or a posterior edge of retinal thickening (or of hard exudates adjacent to the retinal thickening) 500 μm or less from the foveal center; best corrected visual acuity score of at least 55 letters on the ETDRS chart (approximately equivalent to 20/80 or better).	PKC412	50, 100, 150mg/d; 12 months	Orally administered PKC412 at doses of 100 mg/d or higher may significantly reduce macular edema and improve visual acuity in diabetic subjects	(115)
PKC-DRS Study Group 2005	252	60 (8mg/d) 64 (16mg/d) 67 (32mg/d)	61	an Early Treatment Diabetic Retinopathy Study (ETDRS) retinopathy severity level between 47B and 53E inclusive (moderately severe to very severe nonproliferative diabetic retinopathy [NPDR]); best-corrected visual acuity of 44 letters using ETDRS visual acuity protocol (Snellen equivalent of 20/125 or better); no history of scatter (panretinal) photocoagulation for DR; no evidence of glaucoma.	RBX	8, 16, 32mg/d;36-46 months	RBX was well tolerated and reduced the risk of visual loss but did not prevent DR progression.	(116)
PKC-DRS2 Group 2006	685	340	345	(1) Early Treatment Diabetic Retinopathy Study (ETDRS) retinopathy levels of 47A and 53E, (2) best-corrected visual acuity (BCVA) score of 45 letters as measured by the ETDRS visual acuity (VA) protocol (20/125 Snellen), (3) no history of panretinal photocoagulation (PRP) for DR, (4) no evidence of glaucoma, and (5) no history of conditions affecting DR progression.	RBX	32mg/d; 36 months	Oral ruboxistaurin treatment reduced vision loss, need for laser treatment, and macular edema progression, while increasing occurrence of visual improvement in patients with nonproliferative retinopathy.	(117)
Sheetez 2011	203	103	100	(1) Early Treatment Diabetic Retinopathy Study (ETDRS) retinopathy levels of 47A and 53E, (2) best-corrected visual acuity (BCVA) score of 45 letters as measured by the ETDRS visual acuity (VA) protocol (20/125 Snellen), (3) no history of panretinal photocoagulation (PRP) for DR, (4) no evidence of glaucoma, and (5) no history of conditions affecting DR progression.	RBX	32mg/d; 24 months	Patients with greatest RBX exposure experienced less SMVL compared with those in the original PBO group	(118)
Sheetz 2013	1028	520	508	diagnosis of type 1 or 2 diabetes, age≥ 18 years, hemoglobin A1c (HbA1c) 11%, and blood pressure < 160/90 (MBCU) or mean arterial pressure 113 (MBDL).	RBX	32mg/d; 36 months	RBX reduces the relative risk of SMVL from DME by 40% to 50%, with a lesser benefit on morphologic severity.	(119)
PKC-DMES Study Group 2007	686	168 (4mg/d) 174 (16mg/d) 168 (32mg/d)	176	type 1 (18%) or type 2 (82%) diabetes mellitus who were aged 22 to 87 years and had a hemoglobin A1c (HbA1c) level of 5.1% to 13.1%	RBX	4, 16, 32 mg/d; 30 months	Although progression to the primary outcome was not delayed, daily oral administration of RBX may delay progression of DME to a sight-threatening stage.	(120)
Strøm 2005	55	17 eyes (4mg/d) 11 eyes (16mg/d) 14 eyes (32mg/d)	13 eyes	the presence of one sixth or more of disc area (DA) of definite retinal thickening within two disc diameters (DD) of the center of the macula and ETDRS severity of retinopathy level ≤47A, as determined by stereoscopic fundus photograph grading	RBX	4, 16, 32 mg/d; 18 months	RBX treatment was associated with a reduction of retinal vascular leakage in eyes that had diabetic macular edema and markedly elevated leakage at baseline.	(121)
Beckman 2002	14	7	7	healthy volunteer	LY333531	32mg/d; 7 days	Inhibition of protein kinase Cbeta prevents the reduction in endothelium-dependent vasodilation induced by acute hyperglycemia in healthy humans *in vivo*.	(122)
Cherney 2012	20	13	7	age >18 years; type 1 DM; glycated hemoglobin (HbA1C) 6% to 10%; nephropathy (defined as urinary albumin to creatinine ratio of >2.1 mg/mmol in men and >2.8 mg/mmol in women); and estimated GFR ≥ 80 mL/min.	RBX	32mg/d; 8 weeks	PKCβ may modulate endothelial function in type 1 DM, the effect may act through non-RAS pathways in humans with DM.	(123)

Ang, angiotensin; DM, diabetic mellitus; DME, diabetic macular edema; DR, diabetic retinopathy; ETDRS, Early Treatment Diabetic Retinopathy Study; RAS, renin-angiotensin system; RBX, ruboxistaurin; SMVL, sustained moderate visual loss.

Because of unsatisfactory safety, general PKC inhibitors have not been widely used in clinical trials. Hence, specific PKC inhibitors have been developed. The PKC inhibitors used in trials on diabetic microvascular complications were mainly PKC-β isoform inhibitors, including RBX and LY333531.

The effect of RBX has been evaluated mostly in patients with diabetic retinopathy. The PKC-DES trial evaluated the effect of RBX in patients with severe to very severe nonproliferative diabetic retinopathy. After 36-46 months of treatment with RBX, 32 mg/d RBX significantly reduced the risk of moderate visual loss compared with placebo and was well tolerated. However, the progression of diabetic retinopathy was not deferred (116). Another study (PKC-DRS2) recruited patients with moderate to severe nonproliferative diabetic retinopathy and evaluated the effect of RBX on vision loss. The trial was conducted for 36 months, and retinopathy status was assessed every 6 months. After treatment with RBX for 36 months, significant alleviation of visual loss was observed in the RBX group. In addition, the need for laser treatment and macular edema progression were also improved in the RBX group in patients with nonproliferative retinopathy (117). The PKC-DES2 group also conducted a 2-year open-label extension of PKC-DES2 and found that patients with the most exposure to RBX experienced less sustained moderate vision loss (SMVL) than those in the original placebo group (118). Moreover, two phase 3 trials (MBDL and MBCU) demonstrated similar results: RBX is effective in preventing SMVL by 50% in comparison with standard care; however, a significant difference was not achieved (P=0.06). Moreover, diabetic macular edema (DME) morphology-related measures did not show a trend in favor of or against RBX (119). PKC-DMES recruited patients with diabetic macular edema. Treatment with RBX for 30 months also did not show a satisfactory result in delaying the progression of sight-threatening DME (120). In contrast, Strøm et al. found that for patients with DME, 18 months of RBX treatment significantly prevented retinal vascular leakage compared to placebo, particularly in those with elevated leakage at baseline (121). In summary, the PKC-β inhibitor RBX did not show beneficial results in ameliorating the worsening of diabetic retinopathy. However, comparing PKC-DES with PKC-DES2, we found that RBX might be effective in patients with moderate to severe nonproliferative diabetic retinopathy, in which treatment with 32 mg/d RBX showed a satisfactory effect after 3 years. In addition, RBX may be a promising treatment for DME patients with elevated retinal vascular leakage.

Clinical trials on DM-induced endothelial dysfunction were also conducted. Beckman et al. conducted a randomized controlled trial that included healthy subjects. LY333531 or placebo was administered for 7 days before vascular function testing. Forearm blood flow was significantly reduced in patients treated with placebo, and impaired endothelium-dependent vasodilation was also observed. Pretreatment with LY333531 recovered blood flow and attenuated the blood flow response to methacholine chloride during hyperglycemia (122). Moreover, it was found that 8 weeks of treatment with 32 mg/d RBX blunted the effect of hyperglycemia on flow-mediated vasodilation in patients with type 1 DM (123). However, as most clinical trials have focused on diabetic retinopathy, more clinical trials, especially those on diabetic nephropathy, are expected to translate the results of these experiments to clinical applications.

## Conclusions and further perspectives

Given the role of PKC in structural and functional changes in diabetic microvascular complications, it might be a promising target in the treatment of diabetic microvascular complications. However, although many studies have revealed the potential effects of PKC inhibition in alleviating the progression of diabetic microvascular complications, only RBX has shown application potential in diabetic retinopathy, and other PKC inhibitors have not shown satisfactory results in clinical trials. Hence, some challenges and limitations should still be noted in studies at present, as discussed below.

First, many *in vivo* studies used inappropriate disease models. Diabetic microvascular complications are induced by uncontrolled blood glucose over a long period, which is not well mimicked in current studies. In *in vivo* studies, DM is mostly induced by STZ intraperitoneal injection, with or without high-sugar and high-fat feed. STZ targets and destroys islet β cells, causing damage to the regulation of blood glucose. Nevertheless, the mechanisms that drive DM in humans are different from the mechanism of STZ-induced DM. Other factors, such as lipids and the genome, affect the development of DM and obvious diabetic microvascular complications. In addition, current experiments focus on certain inbred species, *such as Rattus norvegicus* and *Mus musculus*. Therefore, the experiment cannot perfectly explain the role of different PKC isoforms in diabetic microvascular complications in humans due to homogeneity. Therefore, the authors suggest that more species, or at least more animals in a group, should be considered in studies of metabolic diseases such as DM.

Second, there is still a lack of biomarkers to evaluate the effect and pharmacodynamics of PKC inhibitors. In terms of evaluating the effect and safety of PKC inhibitors for diabetic microvascular complications, clinical outcomes are the only evidence. This leads to a heavier economic burden. Thus, there is an urgent need to explore new biomarkers to reflect the role and bioavailability of PKC inhibitors in clinical trials, so that the application prospects of PKC inhibitors can be foreseen to be easier and less expensive.

Third, there are still few clinical trials on specific PKC isoform inhibitors other than RBX. As mentioned before, general PKC inhibitors showed poor bioavailability and tolerability in trials, which makes them unacceptable. In addition, PKC inhibitors may also affect other protein kinases, which cause unintended results (124). Hence, specific PKC isoform inhibitors are vital in clinical application. Despite the satisfactory results of specific PKC isoform inhibitors in animal experiments, PKC-β is the main focus of clinical trials. Inhibitors of PKC-α and PKC-δ may also be promising in terms of the results observed in experiments. However, only a few clinical trials on cancer used PKC inhibitors such as rottlerin (125), and diabetic microvascular complications are not being studied. More studies on the effect and safety of other PKC inhibitors are expected to be conducted to determine their applicability.

Finally, studies of new PKC inhibitors with increased selectivity and sample sizes are anticipated. PKC, a kinase family, influences many types of cells and contributes to different cell responses. As we have reviewed, the activation of PKC influences different downstream molecules, and inhibiting the activation of PKC prevents the exacerbation of diabetic microvascular complications. Nevertheless, unwanted effects of PKC isoform inhibitors should also be noted. For example, inhibition of PKC-β may rescue aggravated angiogenesis in diabetic retinopathy, but delayed wound healing may also be induced. In addition, current clinical trials on diabetic microvascular complications have recruited low numbers of participants. As we mentioned before, due to high heterogeneity among humans, many factors influence the effect of trials. Patients of different ages and races and with different lifestyles are differentially affected by the bioavailability of drugs (e.g., clopidogrel resistance in Asian individuals). However, the number of multicenter clinical trials with large sample sizes on diabetic microvascular complications is still unknown. Thus, it is also vital to conduct trials with more patients and take into account high heterogeneity across human subjects.

In terms of quantities of experiments and clinical trials, PKC can be thought of as a promising and attractive target in the treatment and prevention of diabetic microvascular complications. However, owing to the effects of PKC isoforms in various tissues and organs, it is necessary to develop new chemicals that are tissue- or organ-specific to better prevent adverse effects. The development of new highly selective and well-tolerated drugs that regulate PKC and ultimately translate into clinical medicine is expected to treat and prevent diabetic microvascular complications.

## Author contributions

DP and LX formed the reference collection, conducted the reference analysis, and wrote the manuscript. MG contributed to the topic conception and made the decision to submit for publication. All authors contributed to the article and approved the submitted version.

## Funding

This work was supported by the project of National Natural Science Foundation of China (Grant number: 81904025, 82104907) and Fundamental Research Funds for the Central public welfare research institutes Grant (Grant number: ZZ13-YQ-016 and ZZ13-YQ-016-C1).

## Acknowledgments

We would like to thank American Journal Experts for language editing.

## Conflict of interest

The authors declare that the research was conducted in the absence of any commercial or financial relationships that could be construed as a potential conflict of interest.

## Publisher’s note

All claims expressed in this article are solely those of the authors and do not necessarily represent those of their affiliated organizations, or those of the publisher, the editors and the reviewers. Any product that may be evaluated in this article, or claim that may be made by its manufacturer, is not guaranteed or endorsed by the publisher.
